# Designing Safer CRISPR/Cas9 Therapeutics for HIV: Defining Factors That Regulate and Technologies Used to Detect Off-Target Editing

**DOI:** 10.3389/fmicb.2020.01872

**Published:** 2020-08-12

**Authors:** Neil T. Sullivan, Alexander G. Allen, Andrew J. Atkins, Cheng-Han Chung, Will Dampier, Michael R. Nonnemacher, Brian Wigdahl

**Affiliations:** ^1^Department of Microbiology and Immunology, Drexel University College of Medicine, Philadelphia, PA, United States; ^2^Center for Molecular Virology and Translational Neuroscience, Institute for Molecular Medicine and Infectious Disease, Drexel University College of Medicine, Philadelphia, PA, United States; ^3^School of Biomedical Engineering, Science and Health Systems, Drexel University, Philadelphia, PA, United States; ^4^Sidney Kimmel Cancer Center, Thomas Jefferson University, Philadelphia, PA, United States; ^5^Center for Clinical and Translational Medicine, Institute for Molecular Medicine and Infectious Disease, Drexel University College of Medicine, Philadelphia, PA, United States

**Keywords:** CRISPR/Cas9, human immunodeficiency virus, off-target, GUIDE-Seq, DISCOVER-Seq, CIRCLE-Seq, BLISS

## Abstract

Human immunodeficiency virus type-1 (HIV-1) infection has resulted in the death of upward of 39 million people since being discovered in the early 1980s. A cure strategy for HIV-1 has eluded scientists, but gene editing technologies such as clustered regularly interspaced short palindromic repeats (CRISPR)/CRISPR-associated protein 9 (Cas9) offer a new approach to developing a cure for HIV infection. While the CRISPR/Cas9 system has been used successfully in a number of different types of studies, there remains a concern for off-target effects. This review details the different aspects of the Cas9 system and how they play a role in off-target events. In addition, this review describes the current technologies available for detecting off-target cleavage events and their advantages and disadvantages. While some studies have utilized whole genome sequencing (WGS), this method sacrifices depth of coverage for interrogating the whole genome. A number of different approaches have now been developed to take advantage of next generation sequencing (NGS) without sacrificing depth of coverage. This review highlights four widely used methods for detecting off-target events: (1) genome-wide unbiased identification of double-stranded break events enabled by sequencing (GUIDE-Seq), (2) discovery of *in situ* Cas off-targets and verification by sequencing (DISCOVER-Seq), (3) circularization for *in vitro* reporting of cleavage effects by sequencing (CIRCLE-Seq), and (4) breaks labeling *in situ* and sequencing (BLISS). Each of these technologies has advantages and disadvantages, but all center around capturing double-stranded break (DSB) events catalyzed by the Cas9 endonuclease. Being able to define off-target events is crucial for a gene therapy cure strategy for HIV-1.

## Introduction

There are approximately 39 million individuals worldwide that are infected with human immunodeficiency virus type-1 (HIV-1). Combination antiretroviral therapy is effective with respect to suppressing viral load, but it does not remove integrated provirus from the latent reservoir and has not resulted in an effective cure for HIV-1 infection. One of the recent innovative approaches to targeting the latent HIV-1 reservoir has involved the use of the clustered regularly interspaced short palindromic repeats (CRISPR)/CRISPR-associated protein 9 (Cas9) gene editing system that is capable of removing and/or inactivating integrated provirus. Previous studies of CRISPR/Cas9 treatment of a number of HIV-infected cell lines cultured *in vitro*, cells from HIV-1-infected patients cultured *ex vivo*, several small animal models, and animals treated with CRISPR/Cas9 in conjunction with LASER ART have been performed and have demonstrated a proof-of-concept in this regard (Kaminski et al., 2016a,b; Bella et al., 2018; Dampier et al., 2018; Dash et al., 2019; Kaushik et al., 2019). In animals that had been treated with LASER ART and CRISPR, there was a 30% “cure” rate. This was determined by the lack of proviral DNA and the absence of virus in viral outgrowth assays (Dash et al., 2019).

**Figure 1:**
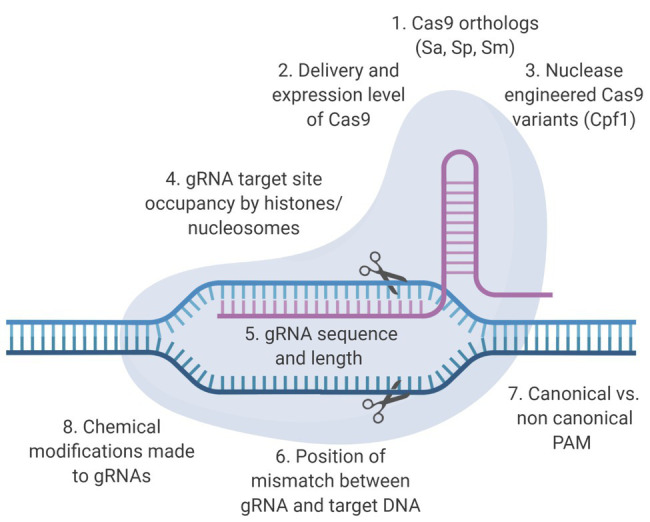
Determinants of on‐ and off-target activity of clustered regularly interspaced short palindromic repeats (CRISPR)/CRISPR-associated protein 9 (Cas9) based therapeutics. The two main components of the CRISPR/Cas9 system are the Cas9 endonuclease and the guide RNA (gRNA). Specific structural alterations of these components have been shown to enhance the on-target excision rate and/or reduce the off-target excision rate. Here, we present some of the factors that influence these excision rates.

**Figure 2:**
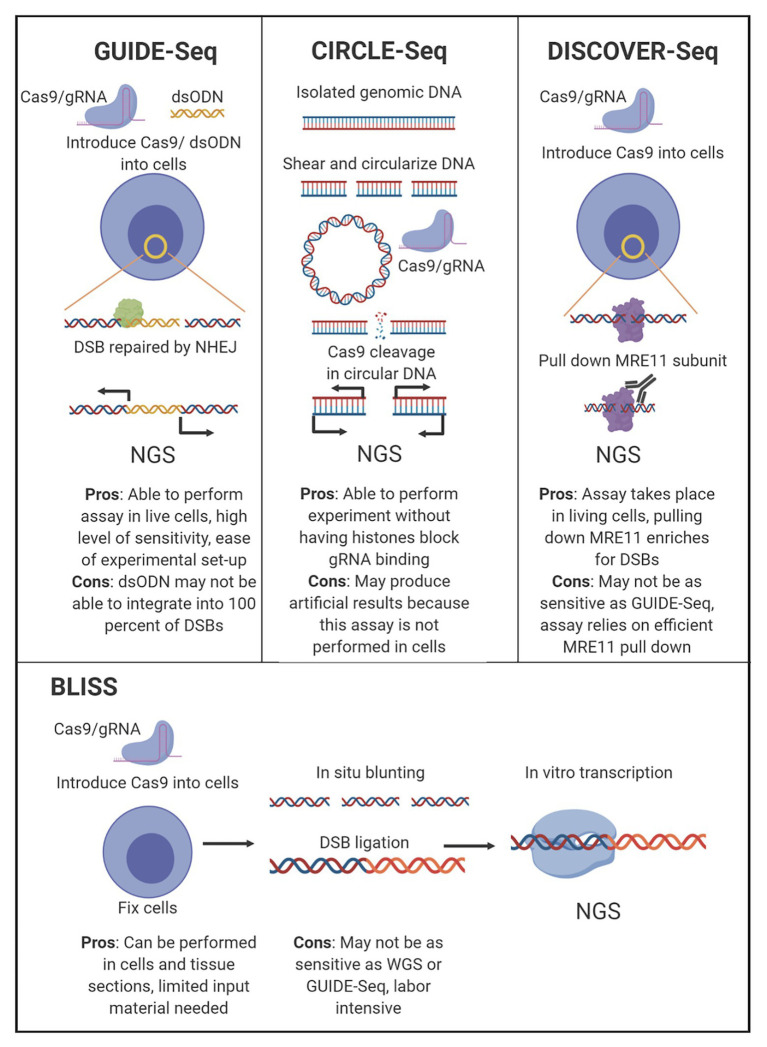
Comparison of leading off-target sequencing methodology. Four of the most widely accepted gRNA off-target sequencing methodologies are depicted with the main experimental steps shown. The major pros and cons for each technology are listed below the indicated technique.

CRISPR/Cas9-based anti-HIV-1 technology has been developing rapidly, but few studies have focused on mitigating the potential for off-target events outside preliminary bioinformatic predictions (Roychoudhury et al., 2018; Darcis et al., 2019) with the exception of whole genome sequencing (WGS). An optimal therapeutic strategy would be both highly effective against the virus and safe for patients. In this context, an effective CRISPR/Cas9 system would be capable of editing the provirus without creating mutations in the human genome. Development of a CRISPR-based anti-HIV-1 therapy within those parameters will require the combined use of bioinformatic, genetic, and functional approaches. This review will discuss the factors that influence the molecular basis of off-target editing (Figure 1), what has been done to characterize off-target promiscuity in the context of RNA-guided targeting of integrated HIV-1 proviral sequences, and why a clearly defined bioinformatic approach with a robust experimental validation protocol to identify off-target potential should be one of the major objectives in the development of an anti-HIV-1 CRISPR/Cas9-based therapy (Figure 2). Designing effective and safe guide RNAs (gRNAs) for anti-HIV-1 therapy requires an in-depth understanding of how the Cas9 system binds and cleaves its target. The safety profile of anti-HIV-1 gRNAs can be enhanced by Cas9 variants utilizing conserved protospacer adjacent motifs (PAMs), near-complete sequence homology with target sequences, and implementation of unbiased off-target detection methods.

## Factors Influencing Off-Target CRISPR/Cas9 Editing

Most gRNAs are preliminarily screened bioinformatically for potential off-target cleavage using sequence complementarity. For the purposes of this review, we define off-target editing as any CRISPR/Cas9 cleavage that does not occur at the binding site intended by a given investigator for the gRNA, and we will discuss factors outside sequence complementarity that can influence this (Figure 1). Off-target editing events have been shown to occur in some *in vitro* studies (Fu et al., 2013; Kuscu et al., 2014; Wu et al., 2014; Frock et al., 2015; Ran et al., 2015; Tsai et al., 2015, 2017; Wang et al., 2015; Wienert et al., 2019). The recognition of editing sites is mediated by Cas9: gRNA ribonucleoprotein (RNP) complex and the formation of an R-loop structure along the target sites (Szczelkun et al., 2014; Zeng et al., 2018). Site recognition begins by the binding of the Cas9 protein to a PAM; a necessary, but not sufficient, precursor to binding and initiating the formation of the R-loop structure. Once bound to the PAM, the main driving factor of target recognition is the progressive Watson-Crick base-pairing of the gRNA with the bound DNA site in conjunction with R-loop expansion. Previous studies showed that CRISPR-mediated cleavage occurred even with mismatches between gRNA and target DNA. In general, mismatches that were distal to PAM site had minimal effect on cleavage efficiency, which allowed CRISPR-mediated cleavage at off-target sites with similar, but not identical sequences (Fu et al., 2013; Hsu et al., 2013; Mali et al., 2013; Pattanayak et al., 2013). Recent studies have identified a variety of other factors, such as gRNA binding stability and substrate availability that influence the likelihood of off-target editing (Fu et al., 2013; Singh et al., 2015).

## PAM Considerations When Selecting Cas9 Orthologs

Once a gRNA has complexed with a Cas9 protein, Cas9 undergoes a conformational change that allows it to bind DNA. The PAM is a target-adjacent short sequence that initiates Cas9 binding. The CRISPR/Cas9 system has been identified in multiple species of bacteria with orthologs often having different PAM requirements (Makarova et al., 2011; Jiang and Doudna, 2017). For example, *Streptococcus pyogenes* (SpCas9) primarily utilizes NGG as its PAM (Deltcheva et al., 2011; Gasiunas et al., 2012; Jinek et al., 2012; Jiang et al., 2013), while *Staphylococcus aureus* (SaCas9) utilizes NNGRRT (Nishimasu et al., 2015; Ran et al., 2015). While SpCas9 primarily recognizes its canonical PAM NGG, it can also bind non-canonical PAM (NAG) less frequently. Previous studies have broadened the limited range of sequences Cas9 proteins could target by relaxing the PAM recognition specificity. The data showed increased targetable range using modified Cas9 with comparable off-target effects (Kleinstiver et al., 2015a,b). The result has also shown that the on-target efficiency was maintained after the PAM stringency was altered and relaxed. In addition to the change of PAM stringency, previous attempts in Cas9 engineering have reduced Cas9: target DNA interaction by neutralizing the positive charges in DNA strand binding domain in the Cas9. This conferred a higher requirement for gRNA: DNA homology for cleavage, therefore increased the targeting specificity (Slaymaker et al., 2016). SpCas9-HF1 with enhanced the targeting specificity was achieved by mutating the residues in the Cas9 that conferred hydrogen bond with target DNA (Kleinstiver et al., 2016). However, in the context of anti-HIV-1 therapy, increasing the PAM length and stringency of the exact number of base-pair matches may limit the potential targets across the HIV-1 genome, and thus number of unique viral variants was targeted within and among individuals. Furthermore, an increased PAM stringency may allow simple escape mutants to develop by targeting for variants lacking the PAM site, with more stringent recognition allowing for an easier escape from therapeutic pressure. In the context of HIV therapeutics, in order to minimize the likelihood of escape variants, multiple gRNAs that target strategic areas in the HIV-1 genome (LTR, gag, and pol) should be used.

One interesting consideration of using multiple gRNAs for HIV is that HIV-1-infected cells with more than one copy may result in interchromosomal recombination. However, it should be noted that studies show most cells to have harbored 1–1.5 HIV DNA copies per cell (Pardons et al., 2019), so interchromosomal recombination should be a remote issue. CRISPR/Cas9 cleavage sites are susceptible to translocations (Frock et al., 2015; Tsai et al., 2015; Stadtmauer et al., 2020). Stadtmauer et al. (2020) reported detection of translocations, which decreased over time in edited cell populations. They also reported that translocations were non-random and more likely associated with particular edited loci (Stadtmauer et al., 2020). Several studies have reported that T cells with translocations can exist without showing pathogenic effects (Michie et al., 1992; McLean and Michie, 1995; Georges et al., 1999). Dash et al. (2019) reported no downstream off-target effects in HIV-1-infected humanized mice treated with HIV-targeting CRISPR/cas9. Translocation events are rare and taken together these results suggest that CRISPR-induced translocations may present minimal risk. Perhaps more importantly, translocations are non-random and induction rates are target-specific meaning that gRNA design may be able to mitigate this risk. Several off-target detection methods are suitable for detection of translocation events, including HTGTS and UDiTaS (Frock et al., 2015; Giannoukos et al., 2018). Further experimentation will be necessary to fully characterize the risks posed by CRISPR/Cas9 with respect to translocation events.

## Target Sequence Homology

Multiple bioinformatic tools exist to predict potential off-target sites using the MIT and cutting frequency determination (CFD) scoring matrices (Hsu et al., 2013; Doench et al., 2016). Sequence homology has been identified as the driving factor in off-target editing events, but full target site-gRNA complementarity is not necessary for Cas9 cleavage to occur. There is variable influence of mismatches at different positions of the gRNA (Hsu et al., 2013; Singh et al., 2015) due to the mechanisms of Cas9 binding (Klein et al., 2018). For example, there will be a high penalty for a mismatch between the DNA site and the gRNA that is proximal to the PAM sequence due to the progressive nature of CRISPR/Cas9-target site hybridization. This would result in the Cas9 system not binding and cleaving its target. In another case, there could be a mismatch between the site and the gRNA that is distal from the PAM sequence, resulting in a low penalty. In this case, the Cas9 system would be more likely to bind and cleave, even though there is a mismatch. This tolerance to mismatch was used to allow investigators to design gRNAs that target more HIV viral variants (Dampier et al., 2014, 2016, 2018; Sullivan et al., 2019). However, mismatch-position-based off-target prediction has raised concerns due to low predictive accuracy (Doench et al., 2016). For example, the MIT and CFD models are unable to explain non-homologous off-target sites with more than four mismatches between gRNA and detected off-target sites, due to the off-target computational pipelines for these techniques assuming anything with more than four mismatches to be due to other types of DNA damage not specific to cas9 cleavage (Tsai et al., 2015, 2017). Moreover, despite the fact that retroviruses and human endogenous retroviruses, such as HERV-K, have evolved with the human genome and have a high similarity to HIV, they are not the driving factor for off-target editing. The majority of predicted off-target editing sites for current anti-HIV gRNAs are very seldomly seen in regions of HERV integration (Link et al., 2018).

## Factors Affecting Off-Target Cleavage Other Than Sequence Homology

Cas9 expression level is another factor that drives off-target promiscuity (Hsu et al., 2013). When Cas9 is delivered in an expression vector, it is continuously expressed. One way to reduce off-target activity involves the delivery of CRISPR/Cas9 in the form of a RNP complex which effectively edits the intended target, and is then degraded within 24–48 hr (Vakulskas et al., 2018). In a study that addressed this issue in the context of HIV-1, Kaminski et al. (2016c) designed a Tat-regulated CRISPR/Cas9 expression vector to prevent expression of Cas9 in cells not containing an actively transcribing provirus. The study has shown that Cas9 is expressed maximally during viral replication or viral rebound, and expression is silenced when the majority of virus have entered a latent state, to decrease the likelihood of overexpression of Cas9 that could lead to increased off-target editing (Kaminski et al., 2016c). In addition, this mechanism could serve as a potential safety mechanism for the expression of the CRISPR system. This would only allow for Cas9/gRNA expression in cells that are actively transcribing viral RNA.

In addition to Cas9 alterations and changes to gRNA sequence, structural changes to the gRNA can increase specificity and reduce affinity for off-target binding. In particular, reduction of gRNA length and structural covalent modifications to gRNA residues have the potential to reduce off-target cleavage (Fu et al., 2014). Reducing the gRNA length to ~17 bp by removing the three most distal nucleotides from the PAM, which are most tolerant to mismatches, can enhance gRNA specificity, albeit the mechanism of which is still an open subject for debate (Fu et al., 2013; Tsai et al., 2015). Off-target affinity is reduced presumably because it becomes a less tolerable mismatch than a full-length gRNA (Fu et al., 2014). Alternatively, modification of the gRNA backbone by adding 2'-O-methyl-3'-phosphonoacetate to particular residues can reduce the probability of off-target cleavage by reducing the stability of the gRNA and Cas9 complex, thereby increasing the stringency of required complementarity without reducing on-target ability (Ryan et al., 2018). However, utilizing shortening or gRNA modifications have yet to be fully understood through experimentation and therefore have not yet been built back into the commonly used design algorithms. With respect to CRISPR/Cas9 and HIV, the above factors do not seem to be actively investigated.

## Detection of CRISPR-Induced Cleavage

### The Crosstalk Between Functional Assessment and *in silico* Design Pipeline for CRISPR-Induced Cleavage

While little has been done to characterize the off-target effects of anti-HIV-1 gRNAs, there is a clear need for off-target screening reflected in the literature. To date, there have been few studies focused on off-target editing of the CRISPR/Cas9 system targeting HIV-1. Many studies may not detect off-target events because *in silico* predictions did not predict a likely off-target cleavage event. Bioinformatic screening has been a first-line approach providing binding specificity predictions for a given gRNA to a specific target. However, there is a clear need to identify technologies that provide real-time functional analysis of off-target activity for gRNAs during the design process. This approach is critical for the safe implementation of CRISPR/Cas9 systems targeting the latent proviral reservoir across HIV-1-infected patient populations.

### Biased Off-Target Detection

The majority of bioinformatic studies that have performed tandem functional characterization of off-target sites used either a SURVEYOR or T7E1 assay. These assays rely on endonuclease digestion of PCR products from selected chromosomal sites of interest based on *in silico* off-target predictions. The need to preselect sites of interest in order to observe off-target edits in both the SURVEYOR and T7E1 assays introduces an inherent bias in off-target detection. In the context of HIV-1 cure strategies, one study performed assessment of off-target cleavage on eight homologous non-target sites in the human genome and did not detect off-target events (Ji et al., 2016) with other studies using similar methods (Hu et al., 2014; Kaminski et al., 2016a,b). This data have been informative but biased due to selected sites for PCR, which were inferred by *in silico* prediction. Furthermore, our current understanding of the CRISPR/Cas9 system in the context of HIV cure strategies is incomplete. Development of a CRISPR/Cas9 HIV-1 therapy cannot rely on methods that screen for off-target cleavage events based on biased predictions and selective screening of expected off-target sites.

### Unbiased Off-Target Detection

Unbiased methodologies have been implemented to screen anti-HIV-1 gRNAs for off-target editing. Two studies performed WGS after disruption and excision of the HIV-1 provirus from infected cells (Hu et al., 2014; Kaminski et al., 2016b). Using WGS addresses one of the limitations of using the SURVEYOR or T7E1 assay. Unlike WGS, SURVEYOR and T7E1 assays are directed at sites, where off-target cleavage events are expected to occur. However, a drawback of WGS is that it sacrifices depth of coverage across the entire genome in order to encompass a larger search space. Furthermore, off-target editing frequency could be well under 0.1% (Kuscu et al., 2014; Wu et al., 2014; Frock et al., 2015; Ran et al., 2015; Tsai et al., 2015, 2017; Wang et al., 2015; Wienert et al., 2019), meaning 100× coverage with WGS may not be able to detect rare off-target events. Also, due to the low average coverage across the genome, it may be impossible to distinguish sequencing errors from low-frequency CRISPR/Cas9 edits. Beyond WGS, there is a need for unbiased off-target detection with an improved signal-to-noise ratio to methodically identify low-frequency off-target events.

There are several techniques currently in use that detect off-target CRISR/Cas9 cleavage in an unbiased manner without WGS, although all utilize an next generation sequencing (NGS) approach as the final measurement step. These assays have been designed to explore gRNA edits both *in vitro*, circularization for *in vitro* reporting of cleavage effects by sequencing (CIRCLE-Seq), as well as for *in vivo* reporting, genome-wide unbiased identification of double-stranded breaks enabled by sequencing (GUIDE-Seq), break labeling *in situ* and sequencing (BLISS), and discovery of *in situ* Cas off-targets and verification by sequencing (DISCOVER-Seq; Tsai et al., 2015, 2017; Yan et al., 2017; Wienert et al., 2019). In the context of designing HIV therapeutics, understanding the functionality of the CRISPR/Cas9 complex *in vivo* is more impactful than the off-target profiles generated by *in vitro* assays, although both types have utility, and each has advantages and drawbacks (Figure 2). While these unbiased assays are purposed to investigate off-target editing for anti-HIV-1 gRNAs, they have currently not been used in published studies.

CIRCLE-Seq relies on a genome-wide, *in vitro* off-target detection utilizing purified genomic DNA. CIRCLE-Seq accomplishes selective amplification of CRISPR/Cas9 cleavage sites *in vitro* by circularizing sheared genomic DNA *via* intramolecular ligation and removing remaining linear DNA with an exonuclease. Circularized DNA fragments are then treated with CRISPR/Cas9, and those containing gRNA-recognizable targets are subsequently linearized. Linearized DNA fragments containing off-target sequence data can then be prepared for NGS.

While this technique may be useful for high-throughput screening of multiple gRNAs, it is limited by its *in vitro* nature. CIRCLE-Seq is constrained as an *in vitro* method because the Cas9 reaction is performed on extracted genomic DNA without DNA repair enzymes present. The requirement for performing the assay is a starting quantity of 25 μg of genomic DNA for each prepared library, which demands a high initial input of DNA derived from either an HIV-1-integrated cell line or tissue samples from either human or animal subjects. An additional consideration is that with extracted genomic DNA, the performance of HIV-1-specific gRNAs is evaluated in the absence of chromatin architecture. DNA accessibility in the CIRCLE-Seq assay is uniform for all sequences, although chromatin environment has been shown to affect the ability of CRISPR/Cas9 to bind and cleave on‐ and off-target sites (Chung et al., 2019). It is unclear if nucleosomes and higher order chromatin structure will limit CRISPR/Cas9 editing and eliminate HIV-1 from these integration sites. Alternatively, *in vivo* methods in which gRNA targeting, Cas9 cleavage, and detection of cleavage events occur within living cells, offer a more clinically relevant model.

Currently there are three choices for *in vivo* detection of off-target CRISPR/Cas9 cleavage: GUIDE-Seq, BLISS, and DISCOVER-Seq. These three techniques function differently, but share common features that make them superior to other off-target detection methods for screening HIV-1-specific gRNAs. Each technique surveys the entire genome in an unbiased manner. GUIDE-Seq works by incorporating short double-stranded oligodeoxynucleotides (dsODNs) into double-strand breaks (DSBs) caused by CRISPR/Cas9 during the repair process, allowing for selective amplification and sequencing of cleavage sites (Tsai et al., 2015). The methodology of GUIDE-Seq DSB detection has been limited by the requirement of nucleofection of dsODNs, which has not been implemented in an animal model. GUIDE-Seq therefore has been constrained to either cell line samples or *ex vivo* cells from human tissue. However, the nature of GUIDE-Seq has allowed for preservation of the chromatin landscape, enabling more relevant interactions of the Cas9 and gRNAs with chromatin architecture in the cell.

BLISS detects DSBs in fixed cells (Yan et al., 2017). BLISS accomplishes selective amplification of CRISPR/Cas9 cleavage sites in fixed cells by blunting and tagging DSB sites with adapters. The tagged ends of the DSB sites are then amplified by *in vitro* transcription, and the RNA is used to prepare NGS libraries. The advantage of BLISS compared to GUIDE-Seq is that GUIDE-Seq detects DSBs repaired by non-homologous end-joining (NHEJ), but will miss DSBs repaired by other processes. While BLISS accounts for this, it requires fixation of cells to label and prepare detected sites, which means detection is limited to DSBs that exist at the time of fixation, whereas GUIDE-Seq will detect the cumulative set of DSBs induced over time.

DISCOVER-Seq detects the cumulative cleavage over time for all endogenous repair pathways, accounting for the short-comings of GUIDE-Seq and BLISS. Furthermore, DISCOVER-Seq has fewer false-positives than GUIDE-Seq and BLISS. However, in a head-to-head comparison targeting the same VEGFA site, 45% of the sites detected by GUIDE-Seq was missed by DISCOVER-Seq (Wienert et al., 2019). DISCOVER-Seq accomplishes selective amplification of cleavage sites by detecting endogenous DNA repair processes. The meiotic recombination 11 (MRE11) subunit of the MRN complex, which localizes to DSBs including Cas9 cleavage sites can be captured with an antibody for chromatin immunoprecipitation sequencing (ChIP-Seq). The MRE11 subunit has been shown to interface with the regions around the Cas9 cut site, and a commercially available antibody can be used to pull down the MRE11 subunit. Following the pull down, the DNA is then sequenced. Similar to BLISS and GUIDE-Seq, this process can also be used to examine CRISPR-mediated cleavage sites in human or animal cells *ex vivo* and can also be performed on cell lines. Finally, DISCOVER-Seq was demonstrated to work in a mouse model *in vivo* (Wienert et al., 2019). It may have potential as a therapeutic option to target tissues and perform real-time discovery in patient biopsy samples. However, this has not yet been examined.

The advantage of these three techniques over WGS stems from the selective amplification and sequencing of Cas9 cleavage sites during library preparation. These methods are distinct improvements over previously published methods such as ChIP-Seq, which have utilized catalytically inactive Cas9 to detect Cas9 binding sites, resulting in abundant false-positives, while new methodologies have been able to detect Cas9 cleavage events, leading to a reduction in false-positives. Notably, for each of the *in vivo* off-target detection methods discussed, DSB detection is the means of identifying CRISPR/Cas9 targets. Endogenous DSBs are, accordingly, also detected by these methods (Tsai et al., 2015; Yan et al., 2017; Wienert et al., 2019). However, given appropriate controls, endogenous DSBs are readily distinguishable from bona fide off-target cleavage events across the genome. They are unbiased because they detect off-target edits across the full genome without being directed to the expected location of off-target edits by *in silico* predictions. Compared to a WGS approach, selective amplification of cleavage sites improves the signal-to-noise ratio during NGS. CRISPR/Cas9 cleavage screening is genome-wide, but the sequence data collected are specific to sites of interest. Despite these advantages, these assays have complex chemistry that is specific to the Illumina MiSeq that limits the sample level throughput to 5–10 samples per run. In addition, other than WGS and DISCOVER-Seq, none of these techniques have been proven to work in animals. Given this, there remains a need for high-throughput methods in order to adequately screen gRNAs for therapeutic applications. In the context of HIV treatment, genetic variation within and between patients is such that no single gRNA or small definitive set of gRNAs are likely to constitute a cure for all patients. Thus, there is still a need to develop a high-throughput screening method that can evaluate the specificity of many gRNAs and CRISPR/Cas9 variants in living cells.

## Limitations for Patient Use

There are currently no off-target detection methods, which can be implemented directly within a patient. However, several methods are suitable for *ex vivo* detection in human primary cells and could be implemented to screen patients for off-target editing (Tsai et al., 2015; Akcakaya et al., 2018; Wienert et al., 2019). There is an adaptation of CIRCLE-Seq called verification of *in vivo* off-targets (VIVO), which consists of a CIRCLE-Seq screening step followed by targeted amplicon sequencing for the highest-probability off-target sites identified by CIRCLE-Seq (Tsai et al., 2017; Akcakaya et al., 2018). VIVO essentially replaces *in silico* off-target prediction models with an *in vitro* detection method. It has been implemented in a mouse model, but the drawback to this technique is that CIRCLE-Seq has a high false-discovery rate, and the need to choose which targets to validate renders this technique biased in the final readout. Wienert et al. (2019) did a head-to-head comparison of DISCOVER-Seq and VIVO using the promiscuous Pcsk9-gP gRNA and identified 17 bona fide off-target sites, which were identified in the CIRCLE-Seq step of VIVO but were not identified because they were not prioritized for amplicon sequencing (Akcakaya et al., 2018; Wienert et al., 2019). DISCOVER-Seq could also be used to screen patients *via ex vivo* detection in primary cells (Wienert et al., 2019). DISCOVER-Seq was demonstrated to work in a mouse model and MRE11 is a highly conserved DNA repair protein. GUIDE-Seq however, is more sensitive than DISCOVER-Seq (Tsai et al., 2015; Wienert et al., 2019). And GUIDE-Seq presents an unbiased genome-wide survey of nuclease activity, which does not have the bias associated with selecting a subset of off-target sites to validate from the expansive CIRCLE-Seq results. A drawback for GUIDE-Seq is that it can be cytotoxic in some primary cells due to the oligonucleotide transfection step (Wienert et al., 2019). Nonetheless, GUIDE-Seq is overall the most promising option currently available to screen patient samples *ex vivo* because it is the most sensitive method which yields the most clinically relevant data.

## Closing Remarks

State-of-the-art CRISPR/Cas9 therapeutics is promising with respect to HIV. But to move forward in the application of this technology, gRNA design must be meticulously investigated to minimize the risk of an off-target event. This is essentially a computational problem, but bioinformatics necessarily relies on laboratory techniques to generate data on which predictions can be formulated and further relies on experimentation to validate predictions. As we have discussed, there are multiple factors affecting the off-target proclivity of gRNAs, many of which can be manipulated for improvements in CRISPR/Cas9 specificity. Recent advances in methodology have made thorough screening of newly design gRNAs possible. Moreover, these assays will allow researchers to design gRNAs that are capable of precision editing without causing deleterious off-target effects and hopefully leading to a safe, therapeutics strategy for an HIV cure. Interestingly, to date, no literature suggest the initiation of oncogenesis or cellular transformation as the result of the CRISPR system. While different articles have examined off-target events by NGS, the effects of these edits have not been determined. If off-target events are found to occur, and it is likely this will be the case in some instance, this will need to be explored.

Finally, CRISPR/cas9 is known to use NHEJ to repair the cleavage site. NHEJ involves several key players including the Ku70–Ku80 hetero dimer (Ku), DNA-dependent protein kinase catalytic subunit (DNA-PKcs) has a high affinity for Ku–DNA ends and several nucleases and ligases (Chang et al., 2017). Important to HIV cure strategies is understanding if these protein levels differ in different cell types (activated versus resting cells and T-cell versus monocyte-macrophage lineage cells). It is also important to understand all of the proteins involved, as different cell types may have different proteins assisted in repair. What all of these are is still an open question as discussed in (Gallagher and Haber, 2018). As such, future studies should examine these levels in all types of cells as both on‐ and off-target cleavage repair could be impacted.

## Author Contributions

NS, AGA, AJA, C-HC, WD, MN, and BW conceptualized the manuscript, contributed to writing and made critical revisions. All authors contributed to the article and approved the submitted version.

### Conflict of Interest

The authors declare that the research was conducted in the absence of any commercial or financial relationships that could be construed as a potential conflict of interest.
